# Effect of *Aloe vera* and carboxymethyl cellulose‐derived binary blend edible coating on the shelf life of fresh‐cut apple

**DOI:** 10.1002/fsn3.3623

**Published:** 2023-08-16

**Authors:** Mansuri M. Tosif, Aarti Bains, Sanju Bala Dhull, Prince Chawla, Gulden Goksen

**Affiliations:** ^1^ Department of Food Technology and Nutrition Lovely Professional University Phagwara India; ^2^ Department of Microbiology Lovely Professional University Phagwara India; ^3^ Department of Food Science and Technology Chaudhary Devi Lal University Sirsa India; ^4^ Department of Food Technology, Vocational School of Technical Sciences at Mersin Tarsus Organized Industrial Zone Tarsus University Mersin Turkey

**Keywords:** *Aloe vera*, browning, cut apples, edible coatings, food preservation, gums

## Abstract

In recent years, the demand and market for minimally processed fruits are increasing worldwide. Fresh‐cut apples are extremely sensitive to environmental factors including oxygen, temperature, and microorganisms in resulting the browning of apples. Therefore, in this study, different concentration of blended edible‐coating solution was prepared using *Aloe vera* and carboxymethyl cellulose (1:1, 1:2, 2:1, 3:3, 3:2, 4:2, 2:4, 3:4, and 4:3, respectively). Lease particle size (101.74 ± 0.67 nm) of the coating solution was observed with 3% *A. vera* and 2% carboxymethyl cellulose (CMC). Afterward, the shelf life of the apples was evaluated for 10 days at refrigeration condition. Results showed that a significant difference was found in weight loss of coated (6.42%–10.26%) and uncoated apples (8.12%–15.32%) for 2–10 days. Moreover, the titrable acidity of the cut apples increased during the storage time. Rheological data emerged that the viscosity of the coating solution decreases with the increasing temperature from 0 to 50°C. Fourier transform infrared spectroscopy data confirmed the presence of hydroxyl group (–OH), C=O, C–O, and N–H banding in the *A. vera*, CMC, and blend‐coating solution. The blend solution indicated excellent antimicrobial efficiency. Total phenolic content of coated and uncoated apples at 0 day was 737.55 mg GAE kg^−1^ for uncoated and 717.88 mg GAE kg^−1^, respectively. Whereas, aerobic and psychrotrophic bacteria counts for edible coated apples significantly lower than control apples. For coated apples, aerobic and psychrotrophic bacteria counts were 1.59 ± 0.84 and 1.25 ± 0.49 log CFU g^−1^ were 4.26 ± 0.67 and 2.68 ± 0.22 log CFU g^−1^ at 10th day, respectively. Overall, it can be inferred that blend of *A. vera* and carboxymethyl cellulose could be used as a nontoxic potential anti‐browning and antimicrobial component for the enhancement of the shelf life and additional nutritional value of fresh‐cut apples.

## Graphical abstract

This study is based on *Aloe vera* and carboxymethyl cellulose blend solution was prepared as an effective natural edible coating anti‐browning and antimicrobial agent. In this article, we discussed the preparation of blend, their rheological behavior, and the blend solution used for the enhancement of the shelf life of cut apples.

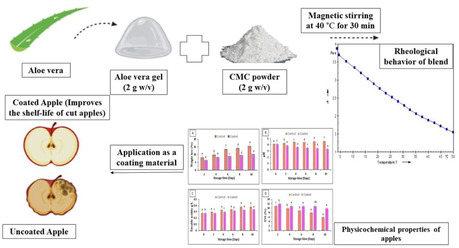

## 1 INTRODUCTION

Minimally processed fruits including apple consist of highly bioactive compounds (flavonoids, phenols, and anthocyanins) which supports the free radical scavenging and antioxidant activity for human health (Duong et al., 2023). Apples processed involved the steps like peeling, cutting, or slicing, to increase the freshness and functional properties of fruits (Aayush et al., 2022; De Corato, 2020). However, cut fruits that received minimum processing are highly susceptible to food spoiling, losing their freshness, and being damaged, squeezed, and destroyed. Additionally, the risk of food spoiling may increase due to the presence of bacteria on the surface of fruits (Alegbeleye et al., 2022; Du et al., 2023). Various preservation techniques such as edible coatings, active atmosphere modification, chemical dipping, and cold storage have been used to maintain the freshness of minimally processed fruits by protecting them from several environmental factors (Blancas‐Benitez et al., 2022; Paidari et al., 2021). Edible‐coated fruit is anticipated to create a modified atmosphere as a result of edible‐coating material act plays a role as a protective barrier material which prevents the food products from various environmental gases. This modified atmosphere, combined with relative humidity and the ideal refrigeration temperature, helps to extend the shelf life for fresh‐cut items (La et al., 2021; Saleem et al., 2021). It can be required to delay physiological activities, such as respiration, to increase shelf life. Thus, the utilization of coatings' ability to alter gas transport may be advantageous for fresh‐cut fruit and vegetables that continue to show active metabolism even during cold storage (Shen et al., 2022). To date, the food industry continues to make extensive use of synthetic antioxidants and artificial chemicals to prevent product oxidation and degradation (Shahbazi, 2018). Researchers examine the use of only natural and biodegradable materials to produce edible films and packaging that are environmentally friendly, healthy, and functional for storage and market distribution to minimize waste and losses (Panahirad et al., 2021). In such cases, natural plant‐derived edible coatings are effectively used to reduce the harmful effect of minimally processed fruits. *Aloe vera* and carboxymethyl cellulose are water‐soluble edible polysaccharides, famous due to their remarkable antimicrobial activity (Arroyo et al., 2020; Riaz et al., 2021; Yousuf et al., 2022). Therefore, they can be potentially used as a natural edible coating to enhance the shelf life of food products by reducing the solutes migration, gas exchange, oxidative reaction rates, and reducing moisture (Xiong et al., 2020). *Aloe vera* gel is receiving increasing attention of researchers due to its affordability, accessibility, and environmental friendliness. Aloin and anthraquinones are just two of the bioactive compounds found in abundance in its gel. It also contains close to 20 amino acids, several vitamins (A, B1, B2, B6, and B12), and is used as a coating material at concentrations ranging from 50% to 100%. In this regard, numerous investigations have been carried out recently by various authors. They used *A. vera* gel as an edible‐coating material for the preservation of grapes (Sarker & Grift, 2021), papaya (Farina, Passafiume, Tinebra, Palazzolo, & Sortino, 2020), apples (Farina, Passafiume, Tinebra, Scuderi, et al., 2020), and mangoes (Ebrahimi & Rastegar, 2020). However, more than one coating material can be used for the enhancement of the antimicrobial efficiency of the coating material. In this regard (Farina, Passafiume, Tinebra, Scuderi, et al., 2020; Farina, Passafiume, Tinebra, Palazzolo, & Sortino, 2020) proved that *A. vera* mucilage‐based edible coatings without additives (lipids and polysaccharides) are less effective than blended edible‐coating solutions (Hajebi Seyed et al., 2021). Similarly, several researchers have been reported to use edible coatings to reduce unfavorable changes caused by minimal processing. They can partially restrict the exchange of gas and vapor, which delays the shrinking of cut produce and modifies the environment around the good. Utilization of polysaccharide blend to minimize the undesirable changes due to several internal and external factors and minimal processing has been studied (Nicolau‐Lapeña et al., 2021; Passafiume et al., 2022; Shah & Hashmi, 2020; Tavassoli‐Kafrani et al., 2022). They have ability to prevent the fruits by improving the barrier, water vapor, and gas exchange properties. Therefore, in this approach, we prepared the coating blend using *A. vera* and carboxymethyl cellulose. Also, characterized the rheological behavior of the blend and physicochemical properties of the coated and uncoated apples were evaluated.

## 2 MATERIALS AND METHODS

### 2.1 Materials

Fresh *A. vera* was harvested from the agriculture farm of Lovely Professional University, Phagwara, Punjab, India. Freshly harvested apples were purchased from the Churag, Mandi, Himachal Pradesh, India. Food‐grade carboxymethyl cellulose (CMC) was purchased from Loba Chem Pvt. Ltd. Sodium hydroxide (NaOH), phenolphthalein indicator, potato dextrose agar, and nutrient agar were procured from the Sigma‐Aldrich Chemic. Folin–Ciocalteu reagent, 2,6‐dichlorophenolindophenol, and 2,2‐diphenyl‐1‐picryl‐hidrazil (DPPH) were purchased from the Otto Chemie Pvt Ltd.

### 2.2 Preparation of *Aloe vera* and CMC blend‐coating solution

*Aloe vera* and CMC blend‐coating solution was prepared according to a method described by Alexandraki et al. (2022). Medium size of *A. vera* was harvested, washed, and the upper layer was removed using a knife. Juice of *A. vera* was prepared using a laboratory blender (Kent, 16044) followed by filtration with a muslin cloth to remove the impurities. For the preparation of the blend solution, different concentrations of *A. vera* and CMC (1:1, 1:2, 2:1, 3:3, 3:2, 4:2, 2:4, 3:4, and 4:3, respectively) solution were prepared in 100 mL of distilled water as shown in Table 1. All the coating solutions were homogenized using high speed homogenizer at 6000 rpm for 10 min followed by centrifugation at 8500 *g* for 15 min coating solution containing lease particle size was selected for the further application. After the preparation of the blend solution for coating treatment, apples (200 g) were washed, peeled, and cut into similar sizes and shapes (square) and stored on the plastic container at refrigerated condition (4–7°C). A few apple pieces were kept as control samples while others were dipped for 2 min into the prepared blended coating solution. Further, control and treated samples were sealed with polypropylene film and stored at refrigerated condition (4–7°C). Storage shelf life was studied for 10 days where microbiological and physicochemical parameters of the coated and uncoated apple were performed.

**TABLE 1:** Different coating formulations of *Aloe vera* (AV) and carboxymethyl cellulose (CMC) blend, their particle size and zeta potential.

Treatments	Concentrations (AV:CMC %)	Average particle size (nm)	Zeta potential (mV)
T1	1:1	201.51 ± 0.84	−14.25 ± 0.84
T2	1:2	188.28 ± 0.42	−19.18 ± 0.68
T3	2:1	185.67 ± 0.75	−10.68 ± 0.52
T4	3:3	199.50 ± 1.05	−22.94 ± 0.38
T5	3:2	101 ± 74 ± 0.67	−15.66 ± 0.88
T6	4:2	154.66 ± 0.71	−28.11 ± 0.15
T7	2:4	166.24 ± 0.33	−24.42 ± 0.36
T8	3:4	1.58 ± 0.88	−24.95 ± 0.55
T9	4:3	1.45 ± 0.64	−37.68 ± 0.43

*Note*: Data shown are the means of three replicates ± standard deviation.

### 2.3 Rheology of the blended solution

Rheology of *A. vera* and CMC blend solution was characterized using the rheometer (Anton Paar, MCR 52, Australia) with a Peltier heating/cooling system and parallel plate layout. To check the rheological behavior, the solution was kept at 52°C for 1 h and the following pre‐shearing procedure was used for the solution prepared in various environments: pre‐shearing at 5 s^−1^ for 10 min, raising the shear rate from 2 to 50 s^−1^ over 3 min, keeping the shear rate constant at 50 s^−1^ for 1 min, and then lowering the shear rate back to 2 s^−1^.

### 2.4 Fourier transform infrared spectroscopy

Fourier transform infrared spectroscopy (FTIR) was used to check the functional groups existing in the *A. vera*, CMC, as well as blend of *A. vera* and CMC. The prepared solutions were dried in tray dryer at 50°C for 24 h and subjected to evaluation of functional groups. FTIR (Perkin Elmer) was used to check the functional group's presence in the blend solution using the infra wave radiations between the mid‐region from 4000 to 400 cm^−1^.

### 2.5 Physicochemical analysis

#### 2.5.1 Weight loss and pH value of apple

Coated and uncoated apples were kept in the pre‐weighted plastic trays at refrigerated condition and weight loss was measured at different time intervals. All the measurements were performed in triplicates. Whereas, the pH of apples was determined according to a method described by Pourdarbani et al. (2022) for different day intervals. For both samples, 30 g of fruits were mixed into 50 mL of distilled water using the high‐speed homogenizer, and pH was measured using a digital pH meter (LAQUAtwin‐pH‐22; HORIBA Scientific).

#### 2.5.2 TSS and acidity of apple

Total soluble solid (TSS) and acidity of the cut apples were evaluated according to method followed by Wang et al. (2023). Apples were homogenized using a laboratory blender with the addition of water and slurry used to determine the titrable acidity and TSS. Then, the filtrate solution was further purified using centrifugation at 4000 *g* for 15 min. A digital reflectometer (Zrs 6060; Prayaag Technologies) was used to check the TSS of apples. Moreover, titrable acidity was checked by titrating the apple juice slurry with 0.1 mol NaOH solution using phenolphthalein as an indicator.

#### 2.5.3 Color value

Based on three variables (L*, a*, and b* value), color was measured using a Hunter colorimeter (Hunter Associates Laboratory, Inc.), according to method followed by Robles‐Sánchez et al. (2013). Herein, the a* value indicates redness and greenness (−80 for green and 80 for red), the *b* value indicates changes from blueness to yellowness, and the *L* value shows lightness (100 for white and 0 for black; −80 for blue and 80 for yellow). The average data from each experiment's three replications were used in the study. Also, the degree of browning was expressed by the *L* value, which shows the lightness of the cut apple.

#### 2.5.4 Firmness

The firmness of the coated and control‐cut apples was analyzed according to a method described by Llano et al. (2016). A texture analyzer (Stable Micro Systems Ltd.) was used to check the firmness of cut apples by applying higher penetration force for a 5 mm diameter probe to penetrate to cut apple piece of 15 mm height to a depth of 15 mm at a rate of 10 mm s^−1^. Five pieces of the cut apples were randomly selected at 0, second, fourth, sixth, eighth, and tenth day and subjected perpendicular to the probe to allow penetration in their geometric center.

#### 2.5.5 Total phenolic content

The Folin–Ciocalteu colorimetric method was employed to determine the total phenolic content of coated and uncoated apples by following the method (Robles‐Sánchez et al., 2013). To initiate the analysis, 100 μL of apple juice were prepared according to the specified pH and titratable acidity and were mixed with 500 μL of Folin–Ciocalteu reagent. Solution was placed in a dark environment for 4 min. Following this, 400 μL of sodium carbonate solution (7.5% w/v) were introduced, and the cuvettes were left undisturbed in darkness at room temperature for 1 h. Absorbance at 760 nm was then measured to ascertain the results. Calibration curve was established using caffeic acid as a standard. The obtained outcomes are presented as the ratio of total phenols content at a specific storage time (TPh) to their initial value (TPh0).

#### 2.5.6 Antioxidant capacity

Antioxidant activity of coated and uncoated apples was evaluated according to method suggested by Robles‐Sánchez et al. (2013), by the 2,2‐diphenyl‐1‐picryl‐hidrazil (DPPH) radical scavenging. Absorbance was measured using spectrophotometer at wavelength of 517 nm. Antioxidant activity was determined using the following equation and expressed as (%) inhibition of DPPH radical.

$$\text{Antioxidant activity}\;\left(\%\right)=\left[\left(\frac{\text{Absorbance of sample}-\text{Absorbance of control}}{\text{Absorbance of sample}}\right)\times 100\right]$$

#### 2.5.7 Ascorbic acid

Ascorbic acid of coated and uncoated cut apples was determined according to method followed by Saleem et al. (2021). Ascorbic acid was performed by titration with 2,6‐dichlorophenolindophenol (DCPIP). It was expressed in mg of vitamin C/100 g cut apples and different concentrations of ascorbic acid were used for the standard curve.

### 2.6 Respiration rate

Respiration rate of the coated and uncoated cut apples were studied according to method suggested by Ali et al. (2013). The effect of coating on CO_2_ was assessed by analyzing the headspace gas composition. Typically, coated and uncoated apple slices (50 g) were placed in a 200 mL tightly sealed glass container at a temperature of 28°C for a duration of 24 h. Throughout this period, headspace samples were periodically withdrawn and subjected to CO_2_ analysis using gas chromatography (Hitachi Model 163). The gas chromatograph was equipped with a thermal conductivity detector and a CTR 1 column (Alltech Associates, Inc.). Helium served as the carrier gas at a flow rate of 40 mL min^−1^. The injector temperature was set at 55°C, the detector at 100°C, and the column temperature was maintained at 55°C. All measurements of respiration rate were performed in triplicate to ensure accuracy and reliability.

### 2.7 Microbiological analysis

Microbial analysis such as the yeast and mold counts, total psychrotrophic counts, and aerobic plate counts of the coated and uncoated apples during the storage at 0, 2, 4, 6, 8, and 10, were determined in triplicate according to method outlined by Mantilla et al. (2013). Fresh and health apple were selected for the microbiological analysis. Herein, apple samples (20 g each) per treatment were homogenized in a sterile stomacher bag. Blended apples material (15 g) was then transferred to another stomacher bag and mixed with 80 mL of buffered peptone water. The mixture was homogenized for 2 min. Subsequently, 10‐fold dilutions were made using this diluent. All counts were performed using Petri films (3M aerobic plate count and 3M yeast and mold count plates, 3M Microbiology, MN). The inoculated 3M aerobic plate count plates (APC) were incubated at 37°C for 48 h. For psychrotrophic counts, the APC plates were incubated at 4°C for 7 days, and all 3M yeast and mold count plates were incubated at 20°C for 7 days. After incubation, colonies were enumerated, and the results were reported as log CFU g^−1^ of sample.

### 2.8 Sensory evaluation

The sensory evaluation of coated and uncoated cut apples in this study was performed by a group of 20 semi‐trained sensory panelists. The evaluators consisted of both male and females between the ages of 25 and 45 years. To reduce any potential bias, the cut apples were presented on serving dishes labeled with anonymous different codes. Sensory characteristics including texture, flavor, appearance, and overall acceptability were evaluated using a 9‐point hedonic scale.

### 2.9 Statistical analysis

All the data are presented as mean ± standard deviation. For statistical data analysis, Microsoft Excel 2021 and SPSS software (version 16; SPSS Inc.) were used to calculate all the results of the experiment. All the experiments were performed in triplicates. We employed one‐way ANOVA and two‐way ANOVA to determine significance difference among the samples.

## 3 RESULTS AND DISCUSSION

### 3.1 Formulation of *Aloe vera* and CMC blend solution

The lease size of blend solution was obtained with concentration of *A. vera* and CMC (3:2), respectively, treatment T5 (particle size: 101 ± 74 ± 0.67 nm and zeta potential: −15.66 ± 0.88 mV) as shown in Table 1. Therefore, coating solution containing lease particle size was further used for edible coating of cut apples. Negative value of zeta potential suggests that *A. vera* and CMC blend solution is highly stable in water and negative charge is due to the presence of several sugars in the *A. vera* and CMC. Larger particle (201.51 ± 0.84 nm) size was observed at the lease concentration of *A. vera* and CMC in T4 (1:1) with zeta potential −14.25 ± 0.84 mV due to higher viscosity of the polysaccharide. Polysaccharide contains monomeric units of the arabinose, xylose, galactose, and rhamnose. These units are having negative charge on their surface thus it has negative zeta potential value and shows excellent stability in water. Furthermore, with increasing the concentration of *A. vera* and CMC particle size was found to be increased in T7 (166.24 ± 0.33 nm) with the concentration of 2:4, respectively. Likewise, nano size of coating showed effective coating barrier for the enhancement of the shelf life of fruits because it has larger surface area with excellent antimicrobial efficiency (Saleem et al., 2021).

### 3.2 Rheological behavior

**FIGURE 1:**
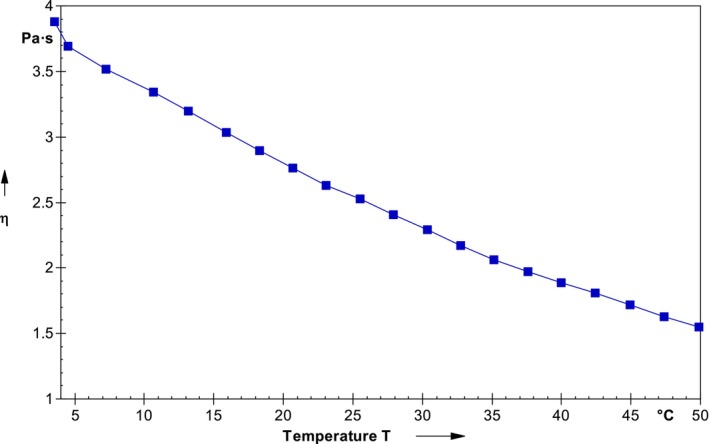
Rheological behavior of the coating solution prepared using *Aloe vera* and carboxymethyl cellulose blend.

Rheological behavior is a key parameter considered for hydrocolloids because stabilizing and thickening properties of the *A. vera* and CMC blend solution are highly dependent upon the temperature, concentration, time of shearing, and shear rate. Figure 1 shows the effect of temperature on the viscosity behavior of the *A. vera* and CMC blend solution for a shear rate of 4 s^−1^. The viscosity of the blend solution decreases with the increasing temperature up to 50°C due to poor heat stability of the polysaccharide (*A. vera* and CMC). Similarly, Cervantes‐Martínez et al. (2014) studied the rheological behavior of *A. vera* and observed the low shear rates with Newtonian behavior of the gel which is similar to our study. Another reason for the decreasing elastic modulus of the blend is due to the loosening of the interconnected group of the polysaccharide hydrogel structure driven by increasing the material's temperature (Medina‐Torres et al., 2019; Nayebi et al., 2020; Saad et al., 2021).

### 3.3 Fourier transform infrared spectroscopy

**FIGURE 2:**
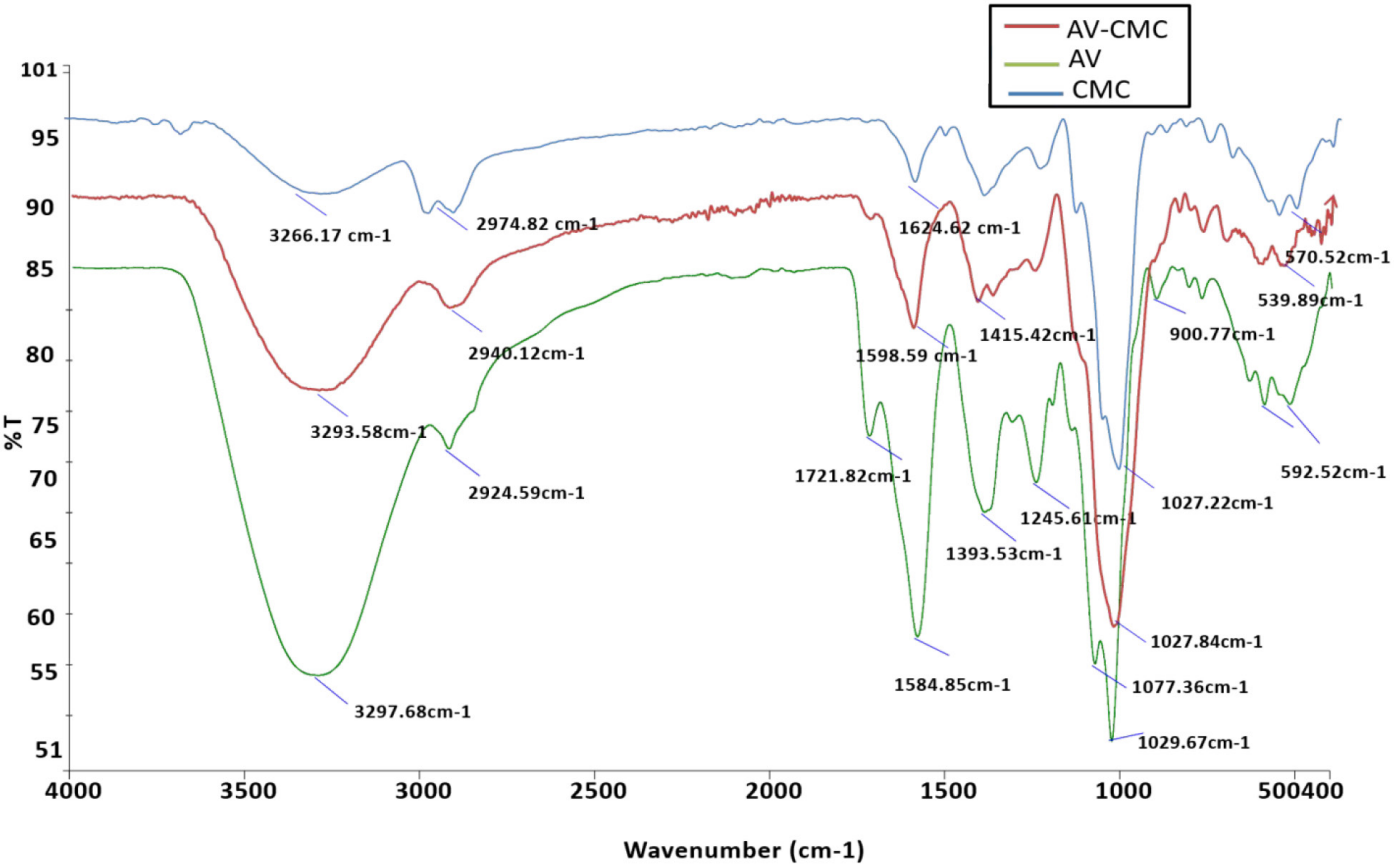
Fourier transform infrared spectroscopy spectra of *Aloe vera* and carboxymethyl cellulose (CMC) blend‐coating solution (AV‐CMC), *A. vera* (AV), and CMC.

FTIR technique was used to study the functional groups existing in the *A. vera*, CMC, and *A. vera*, CMC blend solution (Figure 2). The result of the FTIR showed the five peaks at blend‐coating solution and confirms the stretching vibrations of the hydroxyl (–OH) group at 3293 cm^−1^ *A. vera* 3297.68 cm^−1^ and 3266.17 cm^−1^ for CMC which is due to the presence of organic acids, water, and carbohydrates in polysaccharides (*A. vera* and CMC). Moreover, at band 1598 cm^−1^ for blend solution and 1584.85 cm^−1^ for *A. vera*. Deformation of the hydroxyl group occurred and this band might be due to absorption characteristic band to peptide bonds in polysaccharide C=O (amide I), 1415 cm^−1^ amide‐II (N–H bending) in blend solution due to the presence of minor part of the proteins. Sugars of *A. vera* and CMC were confirmed at 1027 cm^−1^ and for CMC and *A. vera* 1027.22 and 1029.67 cm^−1^ which is attributed stretching of C‐O vibration and related hydroxyl groups. However, this area (800–1300 cm^−1^) is also known as the fingerprinting region of carbohydrates. In this area, the relative intensity was changed which is due to the interaction between amino acids, and hydroxyl groups existing in the *A. vera* and CMC. Similar groups were observed by Andonegi et al. (2020), who used different polysaccharides for the FTIR analysis, and similar interaction was confirmed between two polysaccharides.

### 3.4 Physicochemical evaluation of coated and uncoated cut apples

**FIGURE 3:**
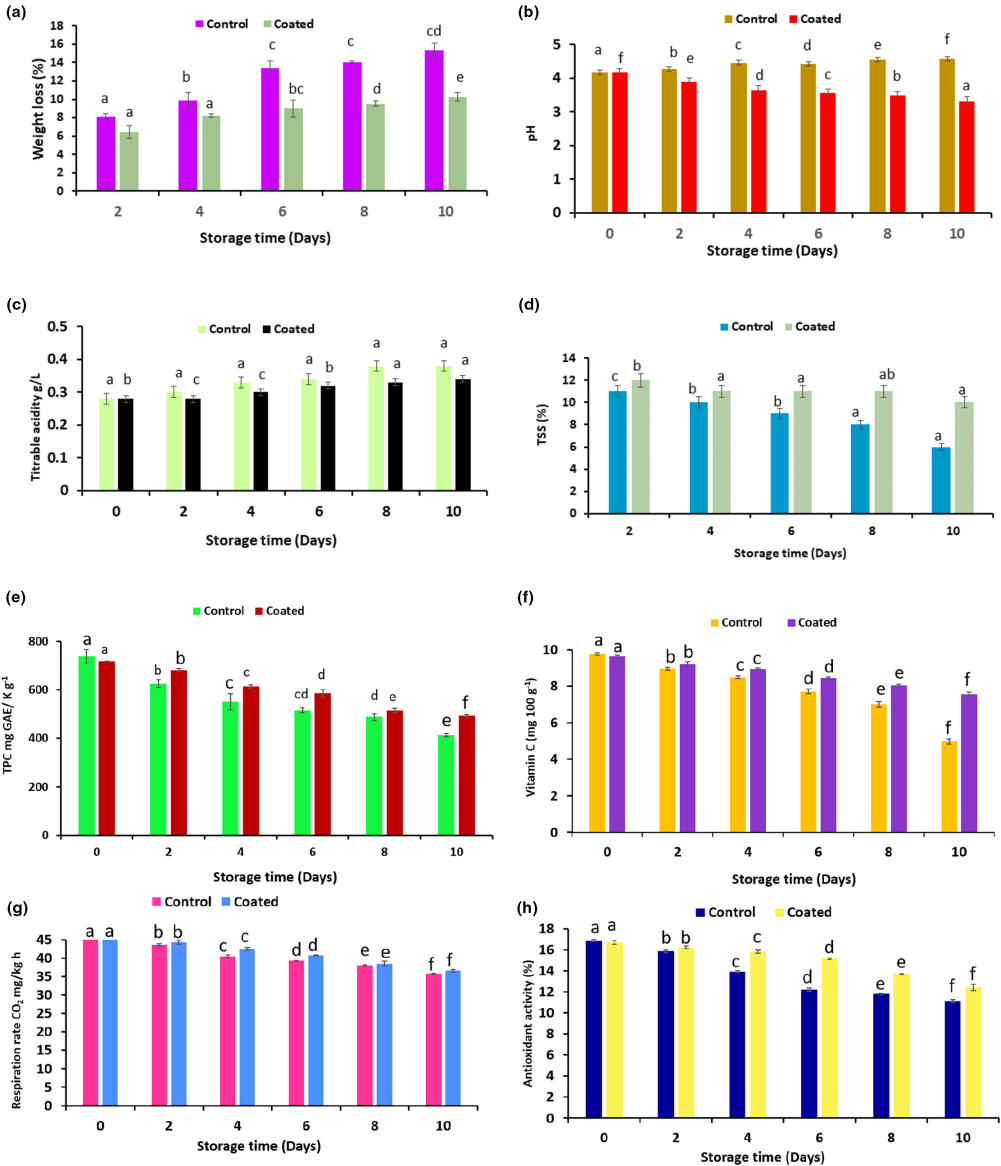
Physicochemical properties of coated and uncoated apples (a) weight loss, (b) pH, (c) titrable acidity, (d) total soluble solid (e) total phenolic content, (f) ascorbic acid, (g) respiration rate, and (h) antioxidant activity (the verticle bars represents the means with SE of two replicate samples, and different lowercase letters above the bars indicate significant difference (*p* ≤ .05) between coating treatment.

The weight loss and pH values of the treated and untreated cut apples are shown in Figure 3a,b, respectively. Herein, it has been observed that the weight loss of *A. vera* and CMC blend‐coated cut apples was significantly lower than an uncoated sample. During the tenth day of the storage study, around 15.32% weight loss occurred for the control and 10.26% weight loss for coated sample. Apple pieces covered with polypropylene films played a significant role in the reduction of weight loss. Similar results were observed by Sapper et al. (2019). Weight loss is a natural occurrence when fruit surfaces are subjected to the elements without protection, which is a factor that directly affects the product's quality. It is expected that applying a surface coating will reduce moisture loss. Therefore, in this study, *A. vera* and CMC blend edible coating showed positive results to reduce the weight loss of cut apples. Similarly, in a study done by Cofelice et al. (2021), they prepared sodium alginate and lemongrass essential oil‐based nano‐edible coatings and checked the effect on cut fruits. However, it has been proven that, by creating a high relative humidity at the surface of the sliced apples, the coating layer certainly appeared to be effective in preventing water loss. On the other hand, minor changes were shown in the pH of both samples. Due to lower humidity, substantial weight loss of cut apples occurred. pH is determined based on the acidic compounds existing in the fruits. Thus, pH and acidity and acidity are the major parameters that decide the freshness of fruits. The acid content of apples tends to decrease over time, most likely as a result oxidation of organic acids during fruit ripening (Cofelice et al., 2021; Feng et al., 2023). Hence, a pH increase is expected during storage time for control (uncoated) samples and decreased for coated samples.

Titrable acidity of the cut apples is expected to increase during the storage time because of the increased respiration rate of the apple based around by peeling, chopping, and other minimal processing activities of the apple (Ballesteros et al., 2022; Panahirad et al., 2021). In our study, minor changes were observed in the titrable acidity of coated and control apples. The variations in acidity were significantly affected by the rate of metabolism due to the respiration of organic acids during storage, acidity typically decreases. Figure 3c shows that the titrable acidity of the edible‐coated apples decreased more slowly (*p* < .05) than control apples because *A. vera* and CMC can modify the internal atmosphere of the apples which can act as a barrier layer. However, titrable acidity was significantly increased during the fourth and sixth due to the production of acids and metabolites by the fungal growth population (Oztuna Taner et al., 2022; Yildirim‐Yalcin et al., 2022). According to Ascencio‐Arteaga et al. (2022), several variables can affect the degree of acidity change, including treatment, variety, and storage conditions. During storage, organic acids could be used as a substitute respiratory substrate, leading to a reduction in acidity. Results of the TSS of coated and control apples are shown in Figure 3d. Herein, it can be observed that *A. vera* and CMC‐coated apples have minor TSS differences than control apples. During the tenth day of storage, the TSS of the control sample was 12% which was decreased to 6% at the tenth day. Whereas, slight changes were observed in the TSS of the coated sample (10% TSS at tenth day).

*Aloe vera* and CMC‐based edible‐coating solutions significantly affected the color value (L*, a*, and b*) of cut apples. Table 2 shows the color values of the control and coated apples. Apples coated with blend solution showed a significant difference in color as compared to control samples during the tenth day of storage. However, the range of the significantly decreased L* value for control from 0 to 10 days is 65.21 ± 0.75 to 34.69 ± 0.15 and for coated sample from 65.81 ± 0.75 to 52.23 ± 0.64, respectively. Also, decreasing in the lightness (L* value) indicates the browning of cut apples (Monteiro Fritz et al., 2019; Morais et al., 2019). Moreover, decreasing in the redness (a* value) suggests an increase in the hue values. Although, a* value was significantly decreased from the zeroth day to the tenth day, from 61.58 ± 0.12 to 48.25 ± 0.88 control, and from 61.63 ± 0.12 to 54.74 ± 0.68 for coated apple. It has been clearly shown that there is a major difference in the color values for uncoated and coated samples as shown in Table 1. The color of cut apples turned into the degradation of pigmented compounds in resulting the white flesh on fruits, due to enzymatic browning (Kahramanoğlu et al., 2019; Nicolau‐Lapeña et al., 2021; Pinzon et al., 2020). During the minimal processing of the cut apples, the polyphenol oxidase (PPO) enzyme is responsible for the browning of the apples, which are liberated from the plant tissues and interact in the presence of oxygen resulting in the formation of a browning compound. On the other hand, prepared coating significantly affected the spoilage of cut apples. Microbial counts in uncoated apples increased during the tenth day of storage (Aloui & Khwaldia, 2016; Pinzon et al., 2020; Sanchís et al., 2016).

**TABLE 2:** The color value of coated and uncoated apples during the storage life.

Color value	Control apple						Coated apple					
	0th day	2nd day	4th day	6th day	8th day	10th day	0th day	2nd day	4th day	6th day	8th day	10th day
L*	65.21 ± 0.75^fA^	44.87 ± 0.26^eA^	42.94 ± 0.43^dA^	38.84 ± 0.02^cA^	36.26 ± 0.25^bA^	34.69 ± 0.15^aA^	65.81 ± 0.75^eA^	61.48 ± 0.62^dB^	59.62 ± 0.35^cB^	56.74 ± 0.36^bB^	55.10 ± 0.41^bB^	52.23 ± 0.64^aB^
a*	61.58 ± 0.12^fA^	58.26 ± 0.15^eA^	54.65 ± 0.68^dA^	52.87 ± 0.98^cA^	50.34 ± 0.12^bA^	48.25 ± 0.88^aA^	61.63 ± 0.12^eA^	60.87 ± 0.74^dB^	58.63 ± 0.45^cB^	56.75 ± 0.12^bB^	55.02 ± 0.32^aB^	54.74 ± 0.68^aB^
b*	9.05 ± 0.85^dA^	8.24 ± 0.25^dA^	7.15 ± 0.44^cA^	6.08 ± 0.25^bA^	5.11 ± 0.64^bA^	4.49 ± 0.41^aA^	9.09 ± 0.85^bA^	9.01 ± 0.65^bB^	8.95 ± 0.82^bB^	7.92 ± 0.65^aB^	7.35 ± 0.55^aB^	6.95 ± 0.59^aB^

*Note*: The color value of coated and uncoated apples during the storage period. Data shown are the means of three replicates ± standard deviation (different lowercase and uppercase letters above the bars indicate significant and non‐significant differences [*p* ≤ .05] between coating treatment).

**FIGURE 4:**
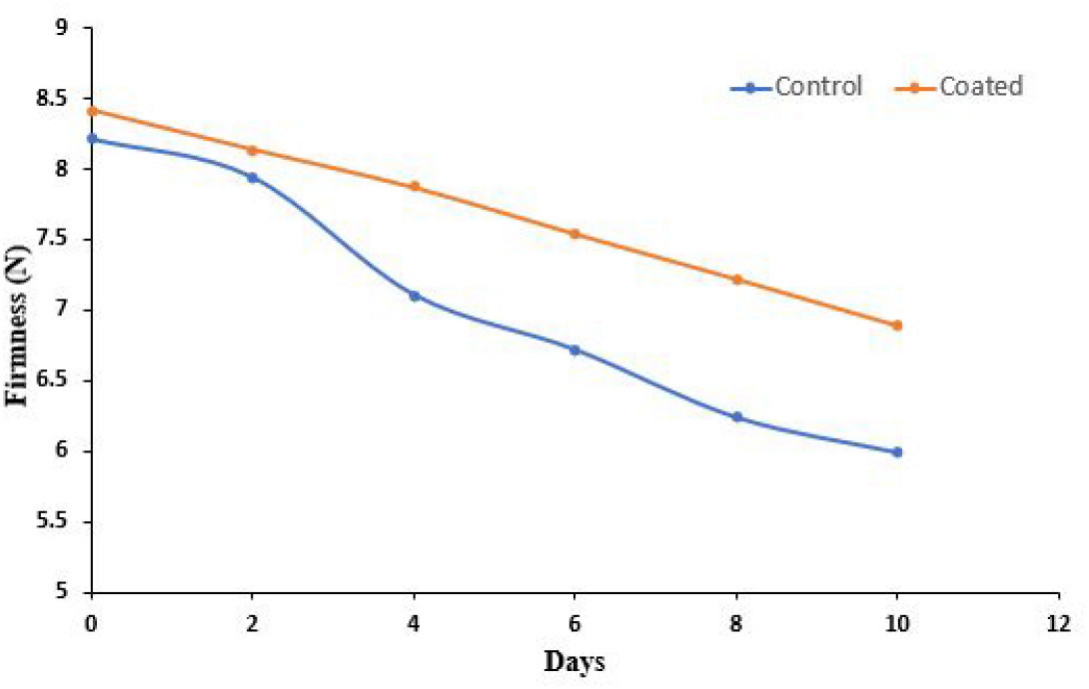
Firmness of the control and coated cut apples during the storage for 10 days.

The firmness value of the cut apple during 10 days is shown in Figure 4. *Aloe vera* and CMC coating showed a significant effect on the firmness of cut apples. Water activity and metabolic changes are major factors affecting the textural loss of fruits and vegetables during storage. The firmness of control apples was decreased from 8.21 ± 0.15 N to 5.99 ± 0.35 N. Softening of the fresh‐cut apples was due to hydrolysis of pectic acid. Whereas, for coated sample, firmness was 8.42 ± 0.09 N at 0 days and 6.89 ± 0.43 N on the tenth day. Herein, *A. vera* and CMC blend can act as a strong protective barrier layer in results increases the shelf life of cut apples. These results are well supported by a study done by Rojas‐Graü et al. (2008). They prepared different polysaccharide coatings using several essential oils for enhancement of the shelf life of cut apples and enhanced the shelf life of cut apples to 23 days.

Total phenolic content of *A. vera* and CMC blend‐coated cut apples (413.40 ± 0.98 mg GAE kg^−1^) were significantly higher than uncoated apples (492.78 ± 0.54 mg GAE kg^−1^) during the tenth day of storage. The effect of *A. vera* and CMC blend on the phenolic content is shown in Figure 3e. It has been shown that, apples coated with blend solution was retain the phenolic content, at 0 day, it was 737.5500 mg GAE kg^−1^ for uncoated and 717.8867 mg GAE kg^−1^ for coated, respectively. Similar results were observed in the study done by Osae et al. (2022). In their study, they coated the tomato fruits and result shows the higher phenolic content in coated tomatoes as compared to uncoated. On the other hand, it has been observed that, coating single component (CMC) decreased in the phenolic content of the cut apples. Whereas, addition of ascorbic acid into the CMC solution increased the phenolic content. Therefore, it has been shown that coating of fruits was effective to maintain the antioxidant activity of the cut fruits or whole fruit. However, edible coated fresh‐cut apples maintained the antioxidant capacity during the first week of storage. Antioxidant activity of the coated apples at zeroth, second, fourth, sixth, eighth, and tenth day were 16.68 ± 0.57, 16.22 ± 0.91, 15.78 ± 0.76, 15.14 ± 0.18, 13.69 ± 0.28, and 12.42 ± 0.69%, respectively. Although, ascorbic acid (Vitamin C) of coated and uncoated apples were decreased during the storage, the application of *A. vera* and CMC blend coatings significantly reduced the loss of ascorbic acid as shown in Figure 3f. Herein, initial ascorbic acid of coated and uncoated apples at 0 day was 9.63 ± 0.50 and 9.7667 ± 0.66 (mg 100 g^−1^) and at 10th day, it was increased up to 7.55 ± 0.10 and 4.98 ± 0.14 (mg 100 g^−1^) for coated and uncoated, respectively. These results are well supported by a study done by Oms‐Oliu et al. (2008). They used different polysaccharides as a coating material for the enhancement the shelf life of fresh‐cut melon. Result revealed the prevention of ascorbic acid, phenolic content, respiration rate, and browning of melon by the edible‐coating material. On the other hand, respiration rate plays important role during the storage of the fruits and vegetables. In our study, edible‐coated apples respiration was significantly reduced from 45.93 ± 0.27 mg kg^−1^ h (0 day) to 36.59 ± 0.34 mg kg^−1^ h (Figure 3g).

### 3.5 Respiration rate

Respiration rate of the cut apples are considered the key indexes for the determination of the storage life of the apples. The average respiration rate of the coated and uncoated cut apples was 36.5967 at 0 days and 35.7933 mg kg^−1^ h at 10th day, respectively. Blend‐coated cut apples showed lower respiration rate than the uncoated apples. Antioxidant activity of the edible‐coated cut apples were significantly increased as compared to uncoated cut apples as presented in Figure 3h. Likewise, Ali et al. (2013) evaluated the respiration rate of the gum arabic‐based tomatoes. Result showed the decrease in the respiration rate of the tomatoes with the higher concentration of the gum arabic. Lower concentration of gum indicated the faster respiration rate as compared to lease concentration.

### 3.6 Microbial analysis

**FIGURE 5:**
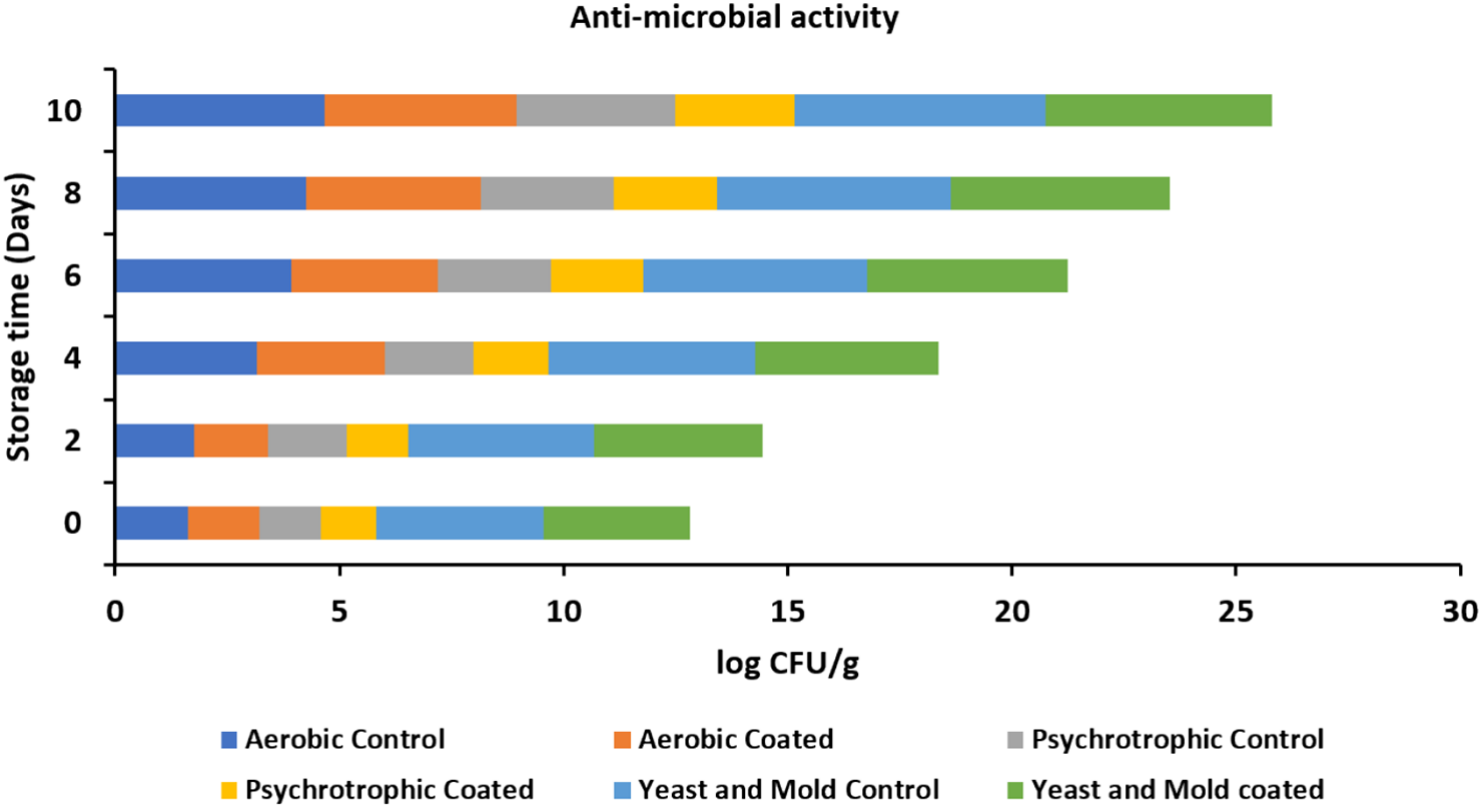
Antimicrobial efficiency of cut apples against bacteria and fungi.

Prepared blended edible coating significantly affected the shelf life of cut apples. Also, microbial counts in uncoated apples increased during the 10 days of storage. Whereas, aerobic and psychrotrophic bacteria counts for edible‐coated apples significantly lower than control apples. For coated apples, aerobic and psychrotrophic bacteria counts were 1.59 ± 0.84 and 1.25 ± 0.49 log CFU g^−1^ were 4.26 ± 0.67 and 2.68 ± 0.22 log CFU g^−1^ at tenth day, respectively (Figure 5). Furthermore, yeast and mold count of the coated and uncoated apples were 5.05 ± 0.36 and 5.58 ± 0.18 log CFU g^−1^ at tenth day, respectively. Toxic chemicals may be created considering the majority of the microorganisms that cause public health concerns, mesophilic bacteria are among the most important microorganisms existing in food. Overall, the result displayed that *A. vera* and CMC can be effectively utilized to reduce the growth of microorganisms in resulting an increment in the shelf life of cut apples (Kumar et al., 2018; Moreira et al., 2015; Song et al., 2013). These results are well supported by a study by Rojas‐Graü et al. (2008) who prepared the edible coatings using various carbohydrate polymers and proteins with different concentrations for the minimally processed apples.

### 3.7 Sensory evaluation

**FIGURE 6:**
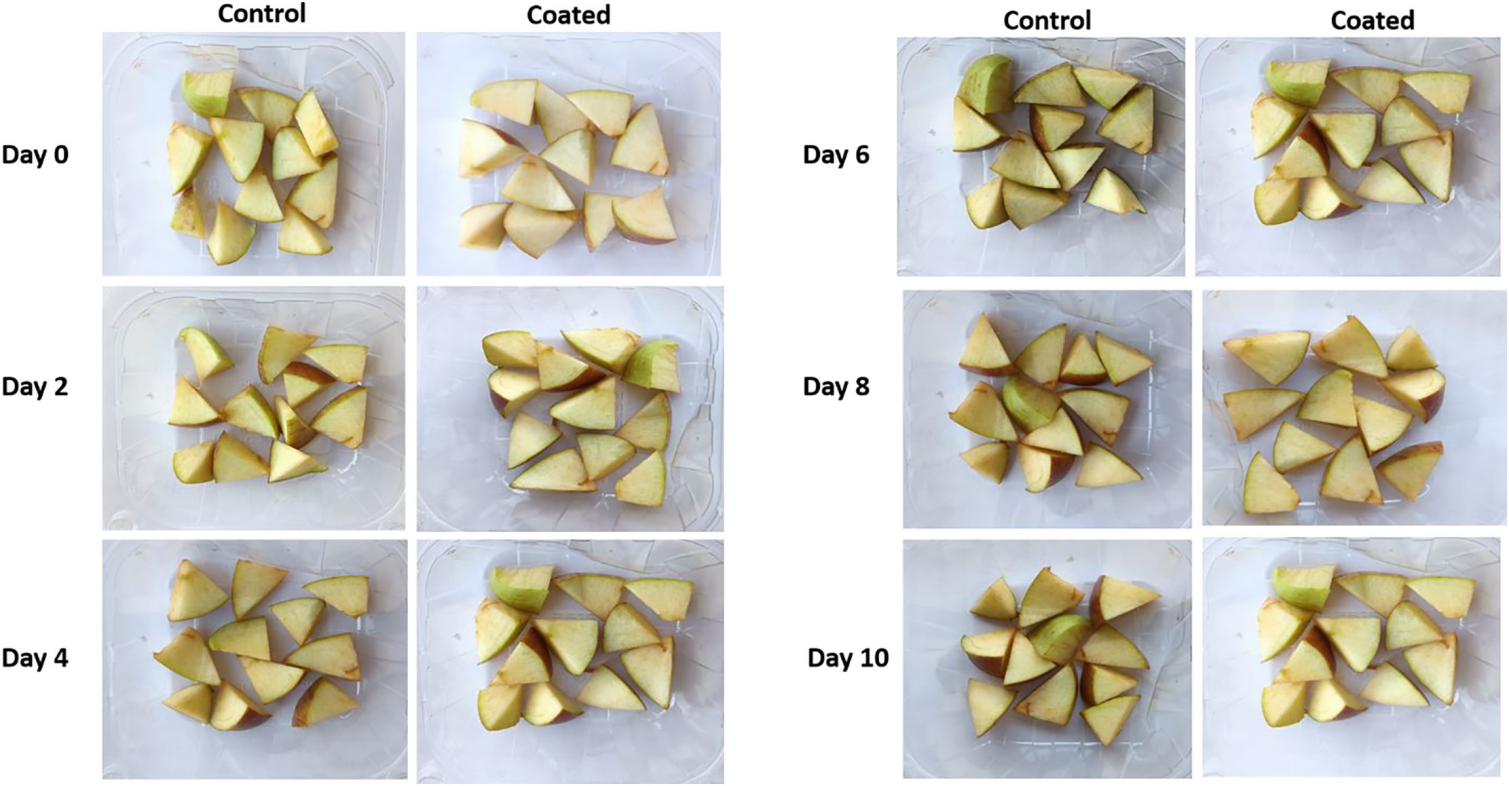
Appearance of coated and uncoated cut apples during the storage.

**FIGURE 7:**
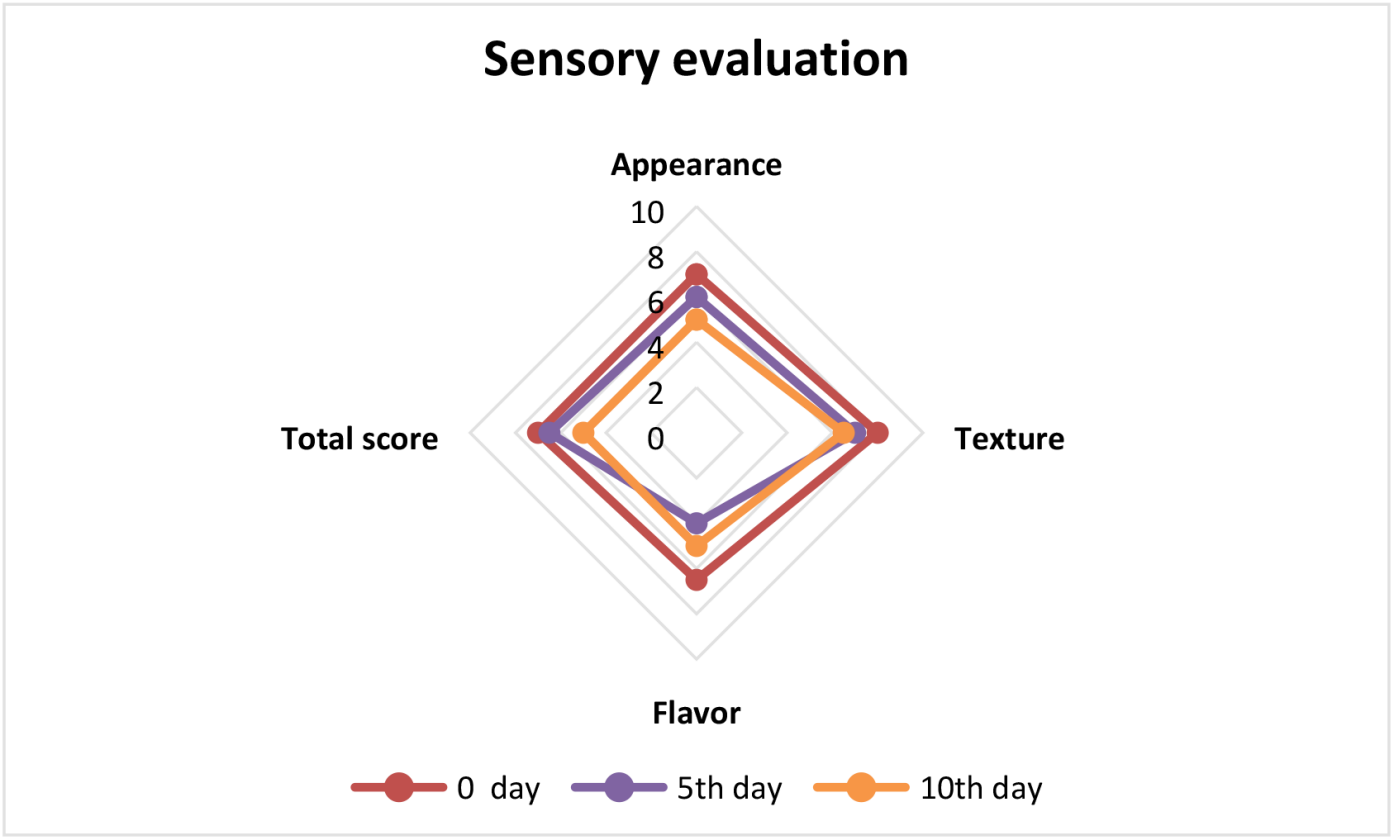
Sensory evaluation of edible coating‐treated cut apples.

Sensory evaluation including texture, appearance, flavor, and total acceptance score is displayed in Figure 6. It can be observed that *A. vera* and CMC blend‐coated cut apples obtained higher score as compared to uncoated apples. While minor difference was observed in the texture of the both samples, uncoated apples received lower score for appearance or color due to the increasing the browning of the apple during the storage period. Overall, panelists resulted that color value of coated sample at fifth day was highly acceptable as demonstrated in Figure 7. Overall acceptance of the edible coated with *A. vera* and CMC blend showed average score of 7 at zeroth day, 6.5 at fifth day, and 5 at tenth day. These might be due to the browning of the fruits takes place with the increasing the storage time. Texture of the cut fruits were also affected by the edible coating and higher score for texture was obtained at day 0 and 5 as compared to day 10.

## 4 CONCLUSION

*Aloe vera* and CMC are water‐soluble edible polysaccharides. Several plant‐derived hydrocolloids are existing and are known as potential edible‐coating materials due to their remarkable functional properties. However, in this study, *A. vera* and CMC coating solutions represented a comparable impact on the edible coating of apples. The coating solution effectively decreased the microbial growth and retain the original color of the apples during storage. Therefore, *A. vera* and CMC‐coated blend can successfully be used to extend the shelf life of cut fruits. The commercial applications of many of these coatings are still very limited, despite the potential advantages of utilizing edible coatings to enhance the quality, shelf life, and safety of fresh and minimal processed fruits, including apples. Additionally, handling and prevention of the cut apples from oxidation (browning) is different in large‐scale production. Thus, this point could be considered as the major limitation of this study.

## AUTHOR CONTRIBUTIONS

**Mansuri M. Tosif:** Conceptualization (equal); data curation (equal); formal analysis (equal); writing – original draft (equal). **Aarti Bains:** Conceptualization (equal); data curation (equal); software (equal); writing – original draft (equal). **Sanju Bala Dhull:** Conceptualization (equal); investigation (equal); methodology (equal); supervision (equal); visualization (equal); writing – original draft (equal); writing – review and editing (equal). **Prince Chawla:** Conceptualization (equal); investigation (equal); methodology (equal); writing – original draft (equal). **Gulden Goksen:** Conceptualization (equal); methodology (equal); supervision (equal); writing – original draft (equal); writing – review and editing (equal).

## FUNDING INFORMATION

No funding was provided for this research.

## CONFLICT OF INTEREST STATEMENT

The authors declare that they have no known competing financial interests or personal relationships that could have appeared to influence the work reported in this article.

## Data Availability

The data that support the findings of this study are available from the corresponding author upon reasonable request.
